# Enbloc resection of the largest thymic liposarcoma: A case report with literature review

**DOI:** 10.1016/j.amsu.2020.09.048

**Published:** 2020-10-03

**Authors:** Samer Alhames, Mike Ghabally

**Affiliations:** a Fellow at The French College of Vascular and Cardiothoracic Surgery; Chief of Thoracic Surgery Department at Saint Louis Hospital, Aleppo, Syria; b University of Aleppo, Faculty of Medicine, Department of Internal Medicine, Division of Cardiology, Aleppo, Syria

**Keywords:** Case report, Liposarcoma, Mediastinal neoplasms, Thymus neoplasms

## Abstract

Liposarcoma is the most common soft tissue tumor which is commonly found in the retroperitoneal region. This kind of tumor is usually well-differentiated with low to no potential to metastasize. Thymoliposarcomas are extremely rare tumors that are difficult to diagnose and differentiate from thymomas and other benign conditions**.**

Presentation of a case:This report presents a case of a 46-year-old male patient with dyspnea, generalized fatigue and non-specific chest pain caused by a giant anterior mediastinal mass. Computed tomography scan revealed a large mass in the anterior mediastinum. CT guided biopsy was consistent with thymolipoma. The tumor was surgically resected. The histological analysis of the tumor revealed thymoliposarcoma.

Discussion:Thymoliposarcoma usually presents with non-specific symptoms. The mean age of the diagnosis is 55.8 years old with a slight predominance in males. The corner stone of the treatment remains surgical excision of the tumor while the role of adjuvant therapy is not well documented.

## Highlights

• Thymoliposarcoma is an extremely rare diagnosis.
• The mean age of diagnosis is 55.8 years with a slight predominance in males.
• The mainstay of treatment remains surgical resection of the tumor.
• The role of adjuvant therapy is not well documented in the literature.

## 1 Introduction

Liposarcoma is most frequently seen in the retroperitoneum; however, it can also develop in unusual sites [1,2]. Thymoliposarcoma is extremely rare type of thymus tumors that was first reported by Havlicek and Rosai in 19,84^3^, and till date, only 10 cases were reported. The diagnosis of thymoliposarcoma can be challenging, as it is hard to be distinguished from other thymus or anterior mediastinal tumors on imaging. Complete surgical resection is the standard treatment; the role of adjuvant therapy is not well established [4].

The presented case in this report is the largest thymoliposarcoma in the literature. The rarity of this tumor brings the importance to such cases to understand its presentation, clinical course and best available treatments. Furthermore, this review represents the first review in the literature of thymoliposarcoma. We believe that this review will help future doctors and researches to conduct further studies in this field.

## 2 Methods

In addition to the presented case, MEDLINE database for all published articles of thymoliposarcoma in the literature is also reviewed. Only liposarcoma involving the thymus gland were included. There was no restriction on language, country or publication date of the paper published. This work is reported in line with SCARE 2020 criteria [5].

## 3 Presentation of case

**Fig. 1:**
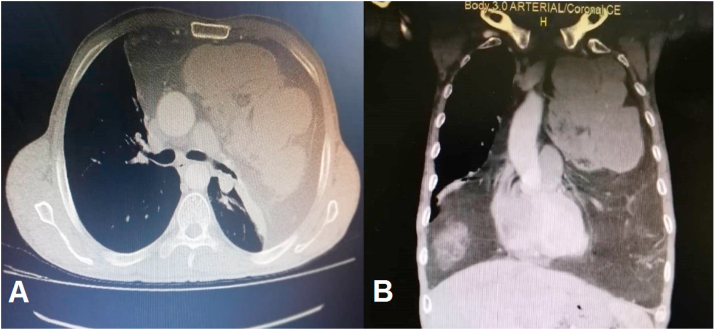
(a) Coronal and (b) sagittal Computed tomography demonstrated an anterior mediastinal tumor with irregular soft tissue density.

**Fig. 2:**
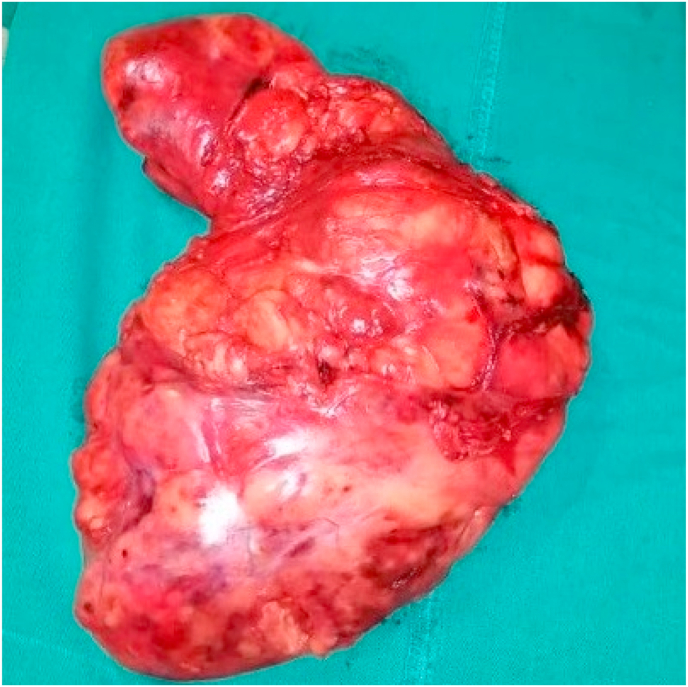
The tumor measuring approximately 35 × 25 cm in size, 5.14 kg in weight.

A 46-year-old male with no significant medical, surgical, psychosocial or family history presented to our institution with a yearlong complaints of fatigue, shortness of breath and non-specific chest pain (see Fig. 1). Part of the work up, the patient underwent chest x-ray, which revealed a mediastinal mass. A follow up computed tomography demonstrated 35 × 25 cm fatty mass occupying the anterior mediastinum causing collapse of the left lung and the upper lobe of the right lung (Fig. 1). CT guided biopsy was performed and was positive for thymolipoma. A thoracic surgeon with 20 years’ experience performed median sternotomy to resect the tumor, a large lobulated capsulated tumor mass was found, the mass was well circumscribed measuring 35 × 25 cm in size and occupying 95% of the left pleural cavity and 50% of the right pleural cavity causing near complete collapse of the left lung. The mass was resected in one piece and it weights 5145 g (Fig. 2). The patient tolerated the procedure well with no complication and minimum blood loss. On gross pathology, the mass showed white and yellow fatty areas of varying sizes and microscopically, the tumor exhibited a mixture of normal-appearing adipocytes mixed with atypical adipocytes. The atypical cells were hyperchromatic, pleomorphic and irregular. In addition, lobules of thymic tissue were embedded in the fatty component of the tumor. Atypical spindle cells were scattered in the adipose tissue and thymic cortex as well. A diagnosis of well-differentiated liposarcoma arising from the thymus was made. Patient was referred for radiation therapy. The patient has returned to his normal life and has been followed up clinically and radiologically since one year with no evidence of recurrence.

## 4 Discussion

Thymoliposarcoma is a very rare mesenchymal tumor that can reach a large size at diagnosis and remains asymptomatic [4]. The tumor usually expands and does not infiltrate into the neighboring structures; thus, most common symptoms are related to nearby structures compression by the tumor mass [1,3,4]. The tumor is usually encapsulated, lobulated with varying proportions of fibrosis and yellow adipose tissue [4]. The differential diagnosis of thymoliposarcoma is that of anterior mediastinal tumor including thymoma, thymolipoma and lymphoma among others [4,6].

Less than 200 cases of primary mediastinal liposarcoma have been reported so far; however, only 10 previous cases involved the thymus and were diagnosed as thymoliposarcomas (Table 1). This case represents the 11th in the literature and largest thymoliposarcoma till date. The mean age of the diagnosis is 55.8 years (range 31–77 years) with a slight predominance in males (male: female ratio 6:4). Most of the cases presented with non-specific symptoms (weight loss, fatigue, non-productive cough and dyspnea). The mainstay of treatment remains surgical resection of the tumor. The role of pre-surgical biopsy is not clear and surgical resection might be challenging given the location of the tumor. Close follow up is recommended. The role of adjuvant therapy (chemotherapy or radiotherapy) is not well documented in the literature with a lack of evidence of its potential role. However, the number of cases is very low and further studies are required in this field. Although thymoliposarcoma in general carry a favorable prognosis, recurrence was reported in three patients in the literature [3,4,8] and distal metastasis were documented in three patients with thymoliposarcoma (Vertebral [3], Nodular [7] and costolateral lung micronudules [2]).

**Table 1:** Thymoliposarcoma cases in the literature (UA: unavailable data; unfortunately we could not provide the full data of the third case).

Case	Age	Sex	Clinical Presentation	Size	Pathology	Treatment	Recurrence	Metastases	Follow up
Havlicek F [3]	39	F	Asymptomatic (pressure sensation)	13 × 9.5 × 5 (650 gr.)	Well differentiated to pleomorphic	Surgical Resection	25 years after the initial surgery (surgery + radiotherapy)	4 years after recurrence (radiotherapy)	32 years
Jones H [7]	1	M	Weight loss, malaise, night sweats, shoulder pain, chest infections	450 gr.	Thymosarcoma with liposarcomatous differentiated	Surgical Resection	21 months of initial surgery (treated with radiotherapy)	Nodular Metastasis	21 months
Cristallini EG [8]	UA	UA	UA	UA	UA	UA	UA	UA	UA
Klimstra DS [6]	72	F	Asymptomatic	No Data	Well-Differentiated	Surgical Resection (after 2 years)	–	–	6 months
Sekine Y [1]	77	F	Asymptomatic	9 × 8.5 × 5.4 cm	Well- differentiated	Surgical Resection + adjuvant radiotherapy	–	–	29 months
Howling SJ [9]	70	F	Non-productive cough	12 cm	Lipoblastic with undifferentiated myxoid pattern	Surgical Resection	–	–	No Data
Sung MT [4]	36	M	Asymptomatic	12.5 × 11 × 5.3 cm	Well-differentiated	Surgical Resection	4 years after surgery (13.2 × 7.5 × 4.4 cm, surgery and radiotherapy)	–	10 months after recurrence
Sivaraman A [10]	55	M	Non-specific chest pain	30 × 12 × 12 (5120 gr.)	Pleomorphic	Surgical with Radiotherapy	–	–	3 months
Mansuet-Lupo A [2]	63	M	Dyspnea, Weight loss	11 cm	Dedifferentiated	chemotherapy (doxorubicin + ifosphamode)	–	Costolateral lung micronodules	No Data
Hosaka Y [11]	63	M	Asymptomatic	8.3 × 5.3 × 8.2 cm	Dedifferentiated	Surgical Resection	–	–	1 year
Alhames S (our case)	46	M	Fatigue, Dyspnea, Non-specific chest pain	35 × 25 (5145 gr.)	Well-differentiated	Surgical Resection	–	–	6 months

## Declaration of competing interest

None.

## Funding

None.

## Consent for publication

Written informed consent was obtained from the patient for publication of this case report and accompanying images. A copy of the written consent is available for review by the Editor-in-Chief of this journal on request.

## Provenance and peer review

Not commissioned, externally peer reviewed.

## Guarantor

The first author is the guarantor of this article
